# Novel Molecular Pathways Elicited by Mutant FGFR2 May Account for Brain Abnormalities in Apert Syndrome

**DOI:** 10.1371/journal.pone.0060439

**Published:** 2013-04-04

**Authors:** Erika Yeh, Roberto D. Fanganiello, Daniele Y. Sunaga, Xueyan Zhou, Gregory Holmes, Katia M. Rocha, Nivaldo Alonso, Hamilton Matushita, Yingli Wang, Ethylin W. Jabs, Maria Rita Passos-Bueno

**Affiliations:** 1 Human Genome Center, Department of Genetics and Evolutive Biology, Institute of Bioscience, University of Sao Paulo, Sao Paulo, SP, Brazil; 2 Department of Genetics and Genomic Sciences, The Mount Sinai Medical Center, New York, New York, United States of America; 3 Department of Plastic Surgery, Faculty of Medicine, University of Sao Paulo, Sao Paulo, SP, Brazil; 4 Department of Neurology, Faculty of Medicine, University of Sao Paulo, Sao Paulo, SP, Brazil; Instituto de Ciencia de Materiales de Madrid - Instituto de Biomedicina de Valencia, Spain

## Abstract

Apert syndrome (AS), the most severe form craniosynostosis, is characterized by premature fusion of coronal sutures. Approximately 70% of AS patients carry S252W gain-of-function mutation in *FGFR2*. Besides the cranial phenotype, brain dysmorphologies are present and are not seen in other *FGFR2*-asociated craniosynostosis, such as Crouzon syndrome (CS). Here, we hypothesized that S252W mutation leads not only to overstimulation of FGFR2 downstream pathway, but likewise induces novel pathological signaling. First, we profiled global gene expression of wild-type and S252W periosteal fibroblasts stimulated with FGF2 to activate FGFR2. The great majority (92%) of the differentially expressed genes (DEGs) were divergent between each group of cell populations and they were regulated by different transcription factors. We than compared gene expression profiles between AS and CS cell populations and did not observe correlations. Therefore, we show for the first time that S252W mutation in FGFR2 causes a unique cell response to FGF2 stimulation. Since our gene expression results suggested that novel signaling elicited by mutant FGFR2 might be associated with central nervous system (CNS) development and maintenance, we next investigated if DEGs found in AS cells were also altered in the CNS of an AS mouse model. Strikingly, we validated *Strc* (stereocilin) in newborn Fgfr2^S252W/+^ mouse brain. Moreover, immunostaining experiments suggest a role for endothelial cells and cerebral vasculature in the establishment of characteristic CNS dysmorphologies in AS that has not been proposed by previous literature. Our approach thus led to the identification of new target genes directly or indirectly associated with FGFR2 which are contributing to the pathophysiology of AS.

## Introduction

Craniosynostosis is a common congenital defect (prevalence of 1∶ 2,500 born alive) characterized by the premature fusion of the neurocranium sutures [1], [2]. Of all the craniosynostosis patients with genetic diagnosis, 32% have mutation in the *FGFR2* (Fibroblast Growth Factor Receptor 2) gene [3]. Among the syndromic craniosynostosis caused by mutation in *FGFR2*, Apert syndrome (AS) and Crouzon syndrome (CS) can be highlighted representing the extremes of the spectrum of clinical variability caused by gain of function mutations in FGFR2

Apert syndrome (OMIM #101200; prevalence of 1∶ 65,000 born alive) is considered one of the most severe forms of craniosynostosis. AS involves the bilateral premature fusion of the coronal sutures along with a midline calvarial bone agenesis, without formation of the metopic and sagittal sutures, and midfacial hypoplasia. Beside cranial defects, patients also have symmetrical syndactyly in upper and lower limbs and true megalencephaly, which allows to distinguish it from other syndromic craniosynostosis [4]. A range of skeletal abnormalities, mental deficiency, central nervous system (CNS) alterations and a variety of visceral malformations were also reported in AS patients [1], [4]. AS inheritance is autosomal dominant and most cases are paternal origin *de novo* mutations [5]. On the other hand, Crouzon syndrome (OMIM # 123500; prevalence of 1∶60,000 born alive) is clinically characterized by craniofacial abnormalities including premature fusion of coronal sutures but no limb or other congenital malformation [6].

AS is mainly caused by the mutations S252W (the most prevalent one, accounting for approximately 64% of the patients) or P253R (33% of the patients) in FGFR2. Both are ligand-dependent gain-of-function mutations which elicit ligand-binding promiscuity of the receptor [7]. This is a distinct molecular mechanism as compared to *FGFR2* mutations found in CS. The CS mutations found in different regions of the gene, constitutively activate FGFR2 by ligand-independent disulphide-mediated covalent receptor dimerization and activation [8].

We have previously described a specific gene expression signature of AS periosteal fibroblasts compared to wild-type (WT) fibroblasts [9]. The differentially expressed genes (DEGs) were associated with cell proliferation, nucleotide metabolism, gene expression regulation, cell adhesion and extracellular matrix organization, and PI3K-MAPK signaling cascades. More importantly, our results together with previous reports suggested that FGFR2^S252W^ over-activates the normal molecular pathways stimulated by WT receptor [9], [10]. Ligand-dependent and ligand-independent gain-of-function mutations in FGFR2 lead to a common bilateral coronal craniosynostosis but to very distinct abnormalities in AS and CS. Thus, we hypothesized that AS mutation in FGFR2 over stimulates downstream signaling pathways activated by WT receptor and further induces pathognomonic novel molecular pathways, which may account for the AS abnormalities not seen in CS.

To test this hypothesis, we performed global gene expression analysis of WT and S252W periosteal fibroblasts stimulated with exogenous FGF2 in order to activate FGFR2 in both groups of cell populations. We also compared the DEGs in response to FGFR2 activation in both groups to CS periosteal fibroblasts harboring mutation C342Y in FGFR2. We finally investigated if DEGs found in this system were also altered in the brain of AS mouse model [11], which would explain CNS abnormalities seen in AS patients.

## Methodology

An outline of the workflow for the performed experiments is shown in Figure S1.

### Subjects

Coronal suture periosteal fibroblasts from three unrelated AS patients, three unrelated CS patients and from three age- and sex-matched control subjects were obtained as previously described [9], [12]. The presence of the S252W and C342Y mutations were confirmed by direct DNA sequencing and expression of the mesenchyme-specific isoform of FGFR2 in the primary fibroblasts was examined by Western Blot and RT-PCR [9], [12]. The project was approved by the Ethical Committee in Research of Human subjects (“Comitê de Ética em Pesquisa - Seres Humanos”) at the Institute of Biosciences University of Sao Paulo. All patients and controls were already enrolled for surgery and treatment by the Department of Plastic Surgery, School of Medicine, University of Sao Paulo, when we contacted them. Thus, those who declined to participate or otherwise did not participate were not disadvantaged in any other way by not participating in the study. Appropriate informed consent was obtained for the donation of the periosteum, a tissue that is usually discarded during surgical treatment, so that this procedure would represent no harm for any of the subjects. Because all the participants were under the age of 18, legal guardians gave written consent on behalf of them.

Care and use of mice for this study were in compliance with the relevant animal welfare guidelines approved by the Johns Hopkins University Animal Care and Use Committee and the Mount Sinai School of Medicine Animal Care and Use Committee.

### Cell Culture

Periostea overlying the coronal suture harvested from AS patients, CS patients or control individuals were used for fibroblast extraction. Primary periosteal fibroblasts derived from periosteal flaps were grown in fibroblast growth medium (DMEM High-Glucose, 20% fetal bovine serum [FBS; GIBCO] and 100 U/mL penicillin and 100 µg/mL streptomycin [1% Penicillin Streptomycin; GIBCO]). Cells were passaged at near confluency with trypsin-EDTA. All cells were cultured in a humidified incubator at 37°C and 5% CO2. All tests were performed between the third and the fifth subcultures.

### Exogenous FGF2 treatment

Periosteal fibroblasts were grown until they reached 80% of confluency. Cells were washed with PBS and then were serum starved for 24 h in DMEM not supplemented with FBS. After this period, control cells were treated with DMEM High-Glucose, 0.5% FBS and experimental cells were treated with DMEM High-Glucose, 0.5% FBS supplemented with recombinant human FGF2 (PeproTech, Rocky Hill, NJ, USA – diluted in 1× PBS –Phosphate Buffered Saline- to a final concentration of 2000 pM) or with DMEM High-Glucose, 0.5% FBS supplemented with 1×PBS. It was reported that similar phosphorylation level of both WT and S252W FGFR2c was observed when treated with 2000 pM of FGF2 [13]. Untreated and treated fibroblasts were harvested after 24 h of addition of FGF2, and had its total RNA isolated and purified as described below. When we first verified the expression level of genes up-regulated by FGFR2^+/S252W^, similar significant alterations in these genes in FGF2 induced control fibroblasts was only observed after 24 h [9].

### Apert Fgfr2^+/S252W^ mice

The Apert Fgfr2^+/S252W^ mice were generated in the laboratory of Dr. Ethylin Wang Jabs [11]. They were inbred on a C57BL/6J background to minimize phenotypic variation due to genetic differences. Genotyping of tail DNA to distinguish mutant from wild-type progeny was carried out by polymerase chain reaction analysis. The primers for *Fgfr2* were as described [11]. Care and use of mice for this study were in compliance with the relevant animal welfare guidelines approved by the Johns Hopkins University Animal Care and Use Committee and the Mount Sinai School of Medicine Animal Care and Use Committee. Mice were killed on P0 by inhalation anesthetics & cervical dislocation and weighed. The carcasses were fixed and whole brains were perfused in RNA later. Our sample consists of two litters inbred in different time, each consisting of two Fgfr2^+/S252W^ and six WT littermates.

### RNA extraction

Cells at a confluency of 80% in 25 cm^2^ cell culture bottles were used for FGF2 treatment followed by microarray and qRT-PCR assays. After a 24 h starvation period S252W and WT fibroblasts were treated with DMEM High-Glucose without FBS supplemented with recombinant human FGF2 (Peprotech) or with DMEM High-Glucose without FBS supplemented with 1×PBS. Total RNA was isolated from FGF2 treated and untreated cells using Nucleospin RNA kit (Macherey-Nagel, Düren, Germany) after 24 h.

Mice whole brain RNA was extracted with RNeasy Mini Kit (Qiagen) following manufacturer's instruction. RNA quality and concentration were accessed by 1.5 percent agarose gel electrophoresis and Nanodrop ND-1000 (Thermo Scientific, Waltham, Massachusetts, USA) respectively.

### Microarray Assays

For each cell line, cDNA was generated with the Affymetrix GeneChip WT cDNA Synthesis and Amplification Kit (Affymetrix, Santa Clara, California) following the manufacturer's instructions. cDNA was fragmented and end labeled with the Affymetrix GeneChip WT Terminal Labeling Kit (Affymetrix, Santa Clara, California). Approximately 5.5 µg of labeled DNA target was hybridized to the Affymetrix GeneChip Human Gene1.0 ST array (Affymetrix, Santa Clara, California)(which interrogates 28869 well-annotated genes) at 45°C for 16 h per manufacturer's recommendation. Hybridized arrays were washed and stained on an Affymetrix GeneChip Fluidics Station 450 (Affymetrix, Santa Clara, California) and scanned on an Affymetrix GCS 3000 (Affymetrix, Santa Clara, California).

Intensity data were subjected to Robust Multichip Average (RMA) and afterwards, to identify differentially expressed genes (DEGs), we used the Limma [14] and Rank -Prod [15] methods, available in the R/Bioconductor package, both with p-value ≤0.05 adjusted by FDR (False Discovery Rate) correction factor. In order to minimize biological variations and focus on the effect of the ligand, we compared the expression data of all three treated fibroblast populations, whether WT or FGFR2^+/S252W^, with the corresponding expression data of the same three untreated fibroblast populations. We extracted the genes that were commonly selected by the two different methods (RankProd and Limma) as significantly differentially expressed (DEGS) in order to minimize false positive occurrence. The Limma method performs statistical analysis similar to that used by SAM (Significance Analysis of Microarrays) [16], and is based on a moderate t-statistics to test the average difference in log expression levels between the treated and the control groups for each gene. The RankProd is a rank-based non-parametric method that uses geometric mean rank for each gene and its distribution is estimated by randomly permuting the observed ranks. The permutation principle partly alleviates the small sample sizes issue, enhancing the robustness against outliers [17]. To analyze the result, we used the IPA software for the analysis of gene interaction and functional classification of DEGs; DAVID for the enrichment of gene ontology and GT (GeneTrail) for analysis of over-or under representation of biological categories and pathways. Analysis of the promoter regions of DEGs was performed through “The IPA Upstream Regulator Analytic” function in IPA. IPA software was also used to study gene interactions and to perform functional classification of DEGs. Briefly, “The IPA Upstream Regulator Analytic” predicts which transcriptional regulators are involved with a set of genes and whether they are likely activated or inhibited.

### Reverse Transcription Reactions and Quantitative Real Time PCR

Complementary DNA (cDNA) was produced from 1 µg of total RNA using Superscript II reverse transcription kit (Invitrogen, Carlsbad, CA, USA). For human fibroblasts, qRT-PCR, assay was performed using approximately 20 ng of cDNA and SYBR Green PCR master mix in an ABI Prism 7500 system (Applied Biosystems, California, USA). For mouse brain qRT-PCR, experiments were run with 20 ng of cDNA and SYBR Green PCR master mix in an ABI Prism 7900 system (Applied Biosystems, California, USA). The PCR conditions for both were: 95°C for 15 s, 60°C for 30 s, and 72°C for 30 s for 40 cycles. In the mouse brain study, first it was performed in a paired 2 WT: 2 S252W littermates sample, if significant difference was observed, sample size was increased to 12 WT: 4 S252W from two litters.

Primers were designed with Primer Express software V.2.0 (Applied Biosystems, California, USA) and the amplification efficiency (E) of each primer was calculated according to the equation: E = 10^(−1/slope)^. The expression data of the studied transcripts was determined by relative quantification in comparison to endogenous controls (human controls: *GAPDH*, *HMBS*, *HPRT1* and *SDHA*; mouse controls: *Ywhaz*, *Tbp*, *Tubb5* and *Bm2*). We verified the gene expression stability of endogenous controls through geNorm VBA applet designed for Microsoft Excel. This tool calculates the most stable reference genes from a set of tested candidate reference genes in a given sample panel, and calculates the gene expression normalization factor for each target sample based on the geometric mean of a defined number of housekeeping genes [18]. The expression data is given by the ratio between each transcript ΔΔCt (E^ΔCT^) and a normalization factor. Samples from all cells analyzed previously in Microarray assay were run in technical triplicates, and the threshold suggested by the instrument software was used to calculate Ct. Primers used in this study are summarized in Table S1.

To assess the statistical significance of the correlation between microarray assay data and the qRT-PCR results we used the nonparametric two-tailed Spearman correlation test, with p-values of less than 0.05 considered to be statistically significant.

### TCF19 immunostaining in human fibroblasts

Fibroblasts were fixed in 4% paraformaldehyde in PBS for 20 min at 4°C, permeabilized in 0.05% Triton X-100 in PBS for 5 min. Nonspecific binding was blocked with 10% BSA in PBS for 1 h at room temperature. Cells were incubated with primary antibody against TCF19 (1∶100, Sigma) overnight at 40°C. After several washes, cells were incubated with secondary (1∶100, AlexaFluor 488, Invitrogen) antibodies against mouse IgG tagged with for 2 h at room temperature. Slides were counterstained with DAPI (4′-6-diamidino-2-phenylindole, Sigma). All images in the same set (treatments and controls) were obtained using the same photographic parameters of exposition and speed. Images were captured using the Axiovision 3.0 image analysis system (Carl Zeiss).

### Strc immunostaining in mouse brain

Two anti-stereocilin antibodies were used, one previously described [19] and the other from Sigma (HPA015731). In our analysis they detected the same targets. Each antibody was tried on both paraffin and frozen sections of P0 WT and S252W brains for IHC, and with both chemical (DAB) and immunofluorescent (IF) visualization for the signal. The Sigma antibody was also used on frozen sections at E16.5 of WT and Apert mice, along with an antibody for Fgfr2 (Santa Cruz) on adjacent sections.

## Results

### S252W mutation alters the gene expression in response to FGFR2 activation

When WT FGFR2 was activated by FGF2, we found 79 DEGs, of which 48 were up-regulated and 31 were down-regulated (Table S2). There was an increased expression of genes involved in MAPK (*DUSP6*, *MAP4K4*, *RASA2* and *ITGA2*), PI3K/Akt (*ITGA2*) and Jak-STAT (*IL13RA2*) signaling pathways. The most significant biological categories among these DEGS were cell growth (IPA: p = 0.0002; DAVID: p = 0.017, GT: p = 0.00024) and cell motility (IPA: p = 0, 0002; GT: p = 0.0005). According to IPA analysis, 31 of the 79 DEGs (39.2%) are on a same gene network associated with movement and cell proliferation. Analysis of the promoter region of the DEGs showed enrichment for genes regulated by the transcription factors SP3 (p = 0.0002), SP1 (p = 0.0014), CLOCK (p = 0.0087), STAT3 (p = 0.01).

Upon FGF2 stimulation, S252W fibroblasts significantly altered expression of 55 DEGs, up-regulating 21 genes and down regulating 34 genes (Table S3). Seven (12.7%) of the DEGs were associated with neurological diseases (*BAT3*, *HS6ST1*, *IFI44L*, *RFC3*, *RPS9*, *STRC* and *TCF19*) according to the IPA analysis (p = 0.003). The most significant biological function was biosynthetic processes (IPA: p = 0.01; GT: p = 0.03). Among the 55 DEGS, 9 (16.4%) were assigned to a same network involved in cell death and cell cycle. Analysis of the promoter region of the DEGs showed enrichment for genes regulated by the transcription factors IRF7 (p = 0.0057), IRF1 (p = 0.0057) and CDKN2A (p = 0.01).

Comparison between the DEGs list for WT and S252W fibroblast showed an overlap of only 5 genes, namely *PRY*, *CYP51A1*, *ARHGAP22*, *ZNF714* and *BDP1*, which corresponded to approximately 8% of the DEGs. Thus, our analysis revealed that the majority of the modifications in gene expression in WT and S252W fibroblasts following FGFR2 activation by FGF2 were different.

### Validation of global gene expression analysis

**Figure 1:**
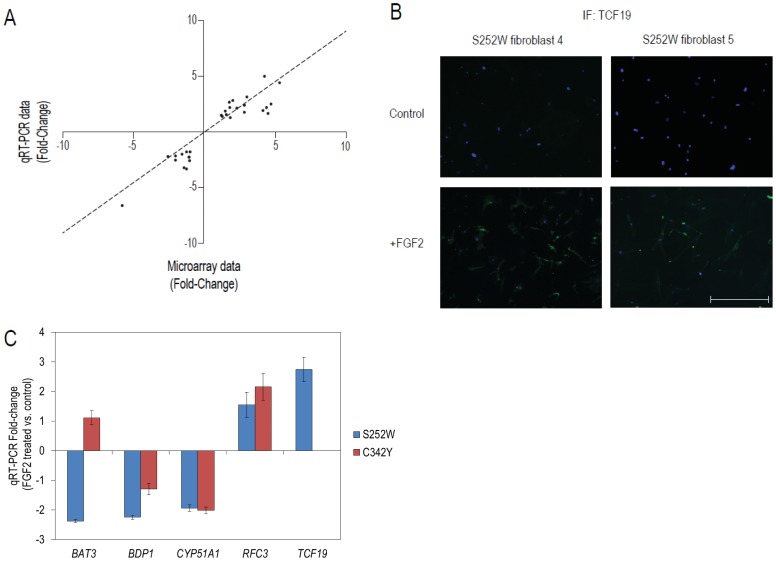
Human periosteal fibroblast experiments. (A) Validation of differentially expressed genes showing the correlation between fold-changes obtained from the Affymetrix microarray experiment and the fold-change values for each gene in each cell line. The correlations between the values of microarray and qRT-PCR fold-changes were calculated through Spearman correlation test. (B) Immunofluorescence staining of TCF19 (green) in two lineages of S252W fibroblasts not included in microarray experiment after 24 h treatment with PBS (control) or FGF2. Blue staining refers to nuclei (DAPI), magnification: 10×; scale bar = 500 µm. (C) Fold-change of the mRNA levels of *BAT3*, *BDP1*, *CYP51A1*, *RFC3* and *TCF19* in FGF2 treated C342Y human fibroblasts and S252W human fibroblasts. Note that there was no *TCF19* expression detected in C342Y human fibroblasts.

To validate the gene expression microarray analysis, we conducted qRT-PCR of a set of DEGS identified between FGF2 treatment and control groups. The mRNA expression of *BDP1*, *CYP51A1*, *DUSP6*, *MAP4K4* and *STC1* were tested in WT fibroblasts treated with FGF2; and *BAT3*, *BDP1*, *CYP51A1*, *RFC3* and *TCF19* in S252W fibroblasts treated with FGF2. The differential expression of each gene between FGF2 treatment and control groups was calculated as a fold-change value, and the correlation between these qRT-PCR fold-change and microarray analysis fold-change for each gene in each cell was evaluated by Spearman correlation test (Figure 1A). The correlation between the values of the two analysis in all cell lines and treatment groups was statistically significant (r^2^ = 0.853, p<0.0001). In conclusion, the values obtained from microarrays and qRT-PCR is consistent and therefore the DEGS selected by bioinformatics analysis are representative of the gene expression profiling experiments.

TCF19 was the only transcription factor in the FGF2 induced S252W fibroblast DEG lists and we opted to further examine TCF19 protein levels through immunofluorescence staining in two S252W fibroblasts not previously included in the microarray experiment. TCF19 was only detectable when S252W fibroblasts where treated with FGF2 (Figure 1B). Results are in agreement with global expression investigation, further validating statistical analysis used in Affymetrix experiment.

### S252W and C342Y mutation affects FGFR2 signaling in different manners

To further delineate whether these gene expression circuitry modifications were consequence of altered ligand binding affinity of FGFR2 or of constitutively active intracellular signaling by the receptor, we sought for BAT3, BDP1, CYP51A1, TCF19 and RFC3 (DEGs in S252W fibroblasts treated with FGF2) expression levels in a C342Y fibroblast through qRT-PCR. The correlation analysis of expression values between C342Y fibroblasts and S252W fibroblasts showed no significant correlation (r^2^ = 0.04, p = 0.904) (Figure 1C). These difference in expression levels were confirmed in biological replicates among independently-derived C342Y and S252W fibroblasts.

### CNS related gene Strc has increased expression in the brain of the Apert mouse model

**Figure 2:**
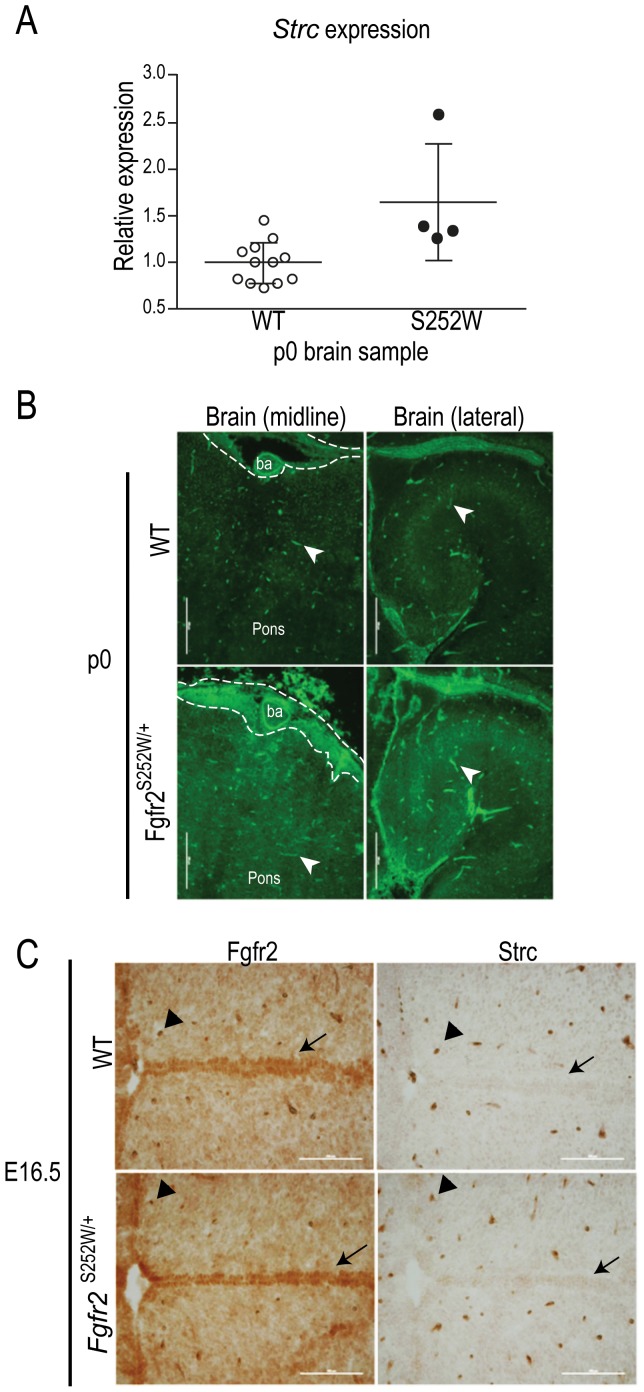
S252W mouse experiments. (A) Quantitative RT-PCR results for CNS related DEG, *Strc*, in p0 Fgfr2^+/+^ (WT) and Fgfr2^+/S252W^ littermates whole brain RNA. (B) Strc immunostaining in the midline and lateral sections of WT and Apert P0 brains. White arrow points to blood vessel cells expressing Strc and dashed lines delimit high Strc expression area in the ventral portion of the pons. ba: basilar artery. (C)Anti-Fgfr2 and the Strc antibody on adjacent frozen sections of E16.5 WT and Apert brains visualized with DAB. Areas of neural tissue expressing Fgfr2 but not Strc are indicated by black arrows. Blood vessels double positive for Fgfr2 and Strc are indicated by arrowheads.

Neuroanatomical abnormalities are a striking phenotype that is part of the wide range of abnormalities that characterize AS and are much more severe than the ones observed in other FGFR2 -associated craniosynostosis [20]. These anomalies are also present in Fgfr2^+/S252W^ mouse model at P0, and did not correlate with patterns of suture closure, suggesting that these alterations are a primary consequence of the mutation [21]. Remarkably, about 13% of the DEGs in FGF2 treated S252W fibroblasts were associated with neurological diseases (*BAT3*, *HS6ST1*, *IFI44L*, *RFC3*, *RPS9*, *STRC* and *TCF19*). Hence we evaluated the expression of the homologues of these 7 genes in P0 Fgfr2^+/S252W^ mice whole brain (Figure S2) together with mRNA levels of the mutant receptor, *Fgfr2*, the epithelial isoform, *Fgfr2b*, the mesenchymal isoform, *Fgfr2c*, and of the *Fgf2* ligand gene as control of the experiment (Figure S2). After analysis of the 7 genes through qRT-PCR, we observed that only one gene, *Strc*, had differential expression in newborn AS mice brain with a 1.6 fold-change (p = 0.006) (Figure 2A).

In order to understand how increased expression of *Strc* could be contributing to AS brain phenotype, we performed immunofluorescent staining of P0 mouse brain. Endothelial cells of blood vessels were the only cells positive for Strc in the p0 brain (white arrows in Figure 2B). This was made more evident by the robust staining observed in the basilar artery that runs ventral to the pons. Moreover, there is a strong positive Strc domain next to the basilar artery, in the ventral portion of the pons (dashed line in Figure 2B) which is wider in the Fgfr2^+/S252W^ compared to WT littermates. Image analysis of 7 sections at different brain levels of Fgfr2^+/S252W^ and WT p0 mouse brain revealed that mutant mice displayed an average of 1.33±0.17 fold more Strc positive blood vessels than control animals. This is in accordance with our previous qRT-PCR results.

We next compared Fgfr2 to Strc expression sites to understand the role of S252W mutation in the overexpression of Strc in the brain. At E16.5, Strc signal did not match the major sites of Fgfr2 expression at the ventricular zones in both Fgfr2^+/S252W^ and WT embryos (arrows in Figure 2C). On the other hand they matched in endothelial cells (arrowhead in Figure 2C). Thus, it is possible that mutant Fgfr2 expressed in endothelial cells may lead to increased Strc expression and affects angiogenesis and vascularization of the developing brain.

## Discussion

Transmission of extracellular signals via plasma membrane proteins to the intracellular compartment is crucial for the cell to recognize and interrelate with neighboring cells and extracellular structures. Fibroblast growth factor receptors (FGFR) mediate the signaling from FGFs into the cell. The amplitude of cell response to FGFR signaling is allowed by both alternate mRNA splicing and binding specificity. In the presence of the S252W mutation, FGFR2 loses normal isoform ligand specificity for most of the ligands. We earlier reported a unique expression profile of S252W coronal suture periosteal fibroblasts and showed that the mutation leads to excessive FGFR2 signaling [9]. Although part of the AS phenotype caused by S252W mutation can be explained by increased downstream signaling, we hypothesized that the mutation also leads to abnormal novel signaling in the cell.

### S252W mutation causes a unique response to FGF stimulation

In order to test this premise, we first performed global gene expression analysis in response to FGFR2 activation in WT or S252W periosteal fibroblasts through microarray experiments. We found that WT fibroblasts stimulated by FGF2 activated the transcription of genes involved in cell proliferation and migration, most particularly those involved with the activation of MAPK and JAK-STAT signaling pathways, all part of the known canonical FGF-FGFR signaling pathway, thus consistent with the extensive literature in this field [22]–[26]. Importantly, even though activated by the same FGF, cells that have the mutant FGFR2 receptor is capable of carrying a different response. This was highlighted by the observation that DEGs of S252W periosteal fibroblasts were not only different from the DEGs of WT fibroblasts, but they were also regulated by a different set of transcription factors. Therefore, activation of the mutant receptor leads to new signaling circuitries that activate different gene regulatory networks.

The only differentially expressed transcription factor activated by S252W FGFR2 was *TCF19*. It has been suggested that TCF19 plays a role in the regulation of expression of other genes necessary for the later stages of cell cycle progression (Ku et al. 1991; Hystad et al. 2007). Since we previously reported that S252W periosteal fibroblasts have enhanced proliferation compared to WT fibroblasts (Fanganiello et al., 2007; Yeh et al., 2011) further experiments will be important to determine its function as a potential mediator of the increase in proliferation in mutant fibroblasts.

Additionally, expression of DEGs in FGFR2^+/C342Y^ fibroblasts treated with FGF2 did not correlate with the expression levels obtained in FGFR2^+/S252W^ fibroblasts under the same treatment. C342Y mutation in FGFR2 (CS) leads to a ligand-independent activation of the receptor [27], while S252W mutation in FGFR2 (AS) leads to an unspecific ligand affinity of the receptor [7], [13]. However, CS patients have milder phenotype compared to AS individuals, which indicates that these two mutations have different molecular and cellular consequences. It is likely that S252W mutation leads to conformational modifications in FGFR2 upon activation, which may affect downstream secondary messenger recruitment. Our results confirm that two different types of gain-of-function mutation in the same gene result in distinct molecular signaling in the same cell type and in the presence of a same ligand.

FGF2 induced differential expression of genes important for development and maintenance of the CNS only in S252W fibroblasts. Of these genes, two – *BAT3* and *RFC3* – were validated through qRT-PCR, attesting the reliability of the microarray assay. These findings are not surprising since the most abundant and widely distributed FGF in the central nervous system is FGF2 [28], [29], which is localized in neurons and glial cells and is expressed in the CNS both during development and postnatally [30]–[32].

### Expression of stereocilin is increased in Fgfr2^S252W/+^ mice brains

Megaencephaly and benign distortion ventriculomegaly are landmarks of Apert syndrome [1], [33]–[36]. Other common CNS alterations observed in Apert syndrome patients are agenesis of the corpus callosum [33], [34], [37], anomalies in limbic structure [33], [35], [37]–[39], and in gyral patterning [33], [37]. Although brain size is not increased in Fgfr2^+/S252W^ mouse model at P0, other CNS anomalies (e.g., asymmetry of cerebral hemispheres and enlarged ventricles) in these animals were found to be highly correlated to the human phenotype [21]. However, molecular signaling that links FGFR2 mutation to these malformations remains unclear.

Given species-specific and tissue-specific differences, we surprisingly found up-regulation of *Strc* (stereocilin) in Fgfr2^+/S252W^ mouse brains, consistent with human S252W fibroblast microarray analysis. Though it is also expressed in brain, eyes, testis and lungs, the role for stereocilin is better established in sensorial hair cells in the cochlea [40]. It is localized at the apical end of kinocilium and is thought to be responsible for the establishment of interaction between stereocilia (specialized motile cilia) and tecta membrane [41]. Loss of function mutations in human *STRC* gene are causative of autosomal recessive deafness [40] and Strc knockout mice also show hearing impairment [19]. It is possible that upregulation of STRC can explain hearing impairment in AS, a deficiency present in more than 90% of the patients [42]. Although no difference in spatial expression of Strc in the brain, there were more Strc positive blood vessels in Apert mice brains in association with FGFR2 expression. Our results indicate that there is an unknown role for Strc in endothelial cells in AS CNS probably through biomechanical forces response.

## Conclusion

Although part of the phenotype caused by S252W mutation can be explained by over-activation of the normal molecular pathways elicited by WT receptor, the mutation also induces novel molecular pathways. This characteristic distinguishes AS from other FGFR2-related syndromic craniosynostosis, such as CS. Moreover, our data suggests that abnormal signaling elicited by mutant FGFR2 induces differential expression of genes important for development and maintenance of the CNS. Among these genes, we validated Strc in newborn Apert mouse brain, suggesting a role for endothelial cells in the establishment of landmark CNS abnormalities of AS. These results also suggest that STRC is in the same circuitry as FGF/FGFR2. Further investigation of the vascularization in the CNS in AS is required for a better understanding not only of the clinical manifestations but also of the role of FGF signaling in brain development.

## Supporting Information

Figure S1 **Experiments workflow.**
(DOCX)

Figure S2 **Quantitative RT-PCR results for CNS related DEGs in p0 Fgfr2^+/+^ (WT) and Fgfr2^+/S252W^ (S252W) littermate whole brain RNA.** A–K shows each of these DEGs.
(DOCX)

Table S1 **Primers used for quantitative real time PCR.**
(DOCX)

Table S2 **Differentially expressed transcripts in FGF2 - treated WT fibroblasts compared to the same samples without treatment.** In each treatment, transcripts are ordered by average Fold change ratio (treated vs non-treated) of the replicates.
(DOCX)

Table S3 **Differentially expressed transcripts in FGF2 - treated S252W fibroblasts compared to the same samples without treatment.** In each treatment, transcripts are ordered by average Fold change ratio (treated vs non-treated) of the replicates.
(DOCX)
