# Supplementary figures and images for: Novel Molecular Pathways Elicited by Mutant FGFR2 May Account for Brain Abnormalities in Apert Syndrome
Source: PLoS One. 2013 Apr 4;8(4):e60439. doi: 10.1371/journal.pone.0060439 (PMC3617104; doi:10.1371/journal.pone.0060439)

Figure S1


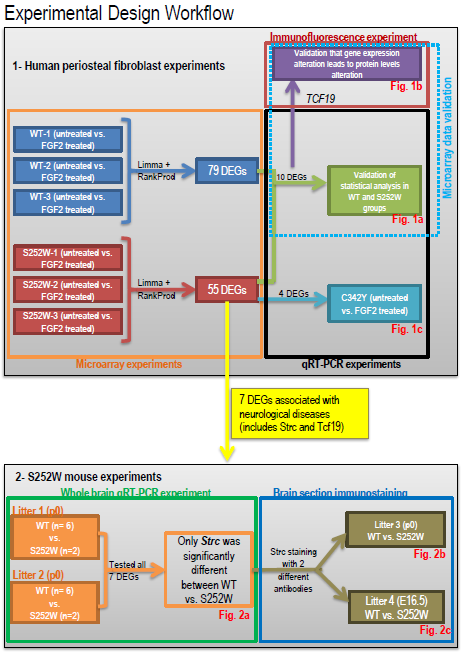

Supplement: Figure S1 — Experiments workflow. (DOCX) [file pone.0060439.s001.docx]

# Figure S2


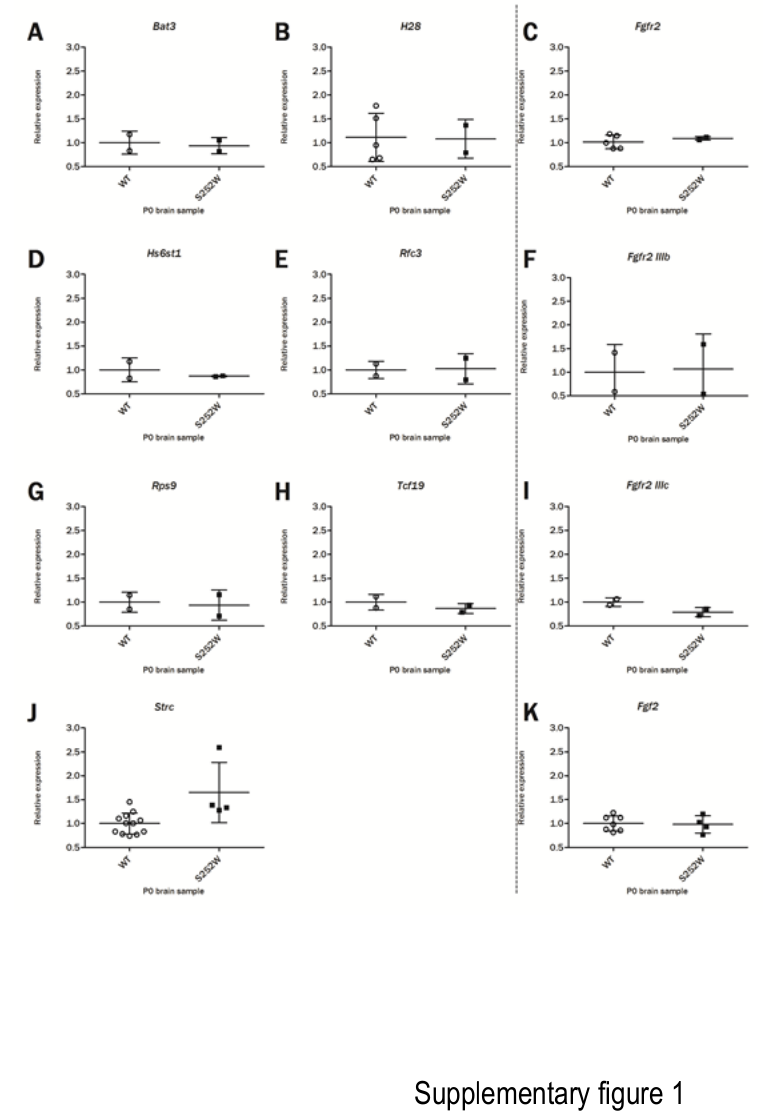

Supplement: Figure S2 — Quantitative RT-PCR results for CNS related DEGs in p0 Fgfr2+/+ (WT) and Fgfr2+/S252W (S252W) littermate whole brain RNA. A–K shows each of these DEGs. (DOCX) [file pone.0060439.s002.docx]

**Table S1**


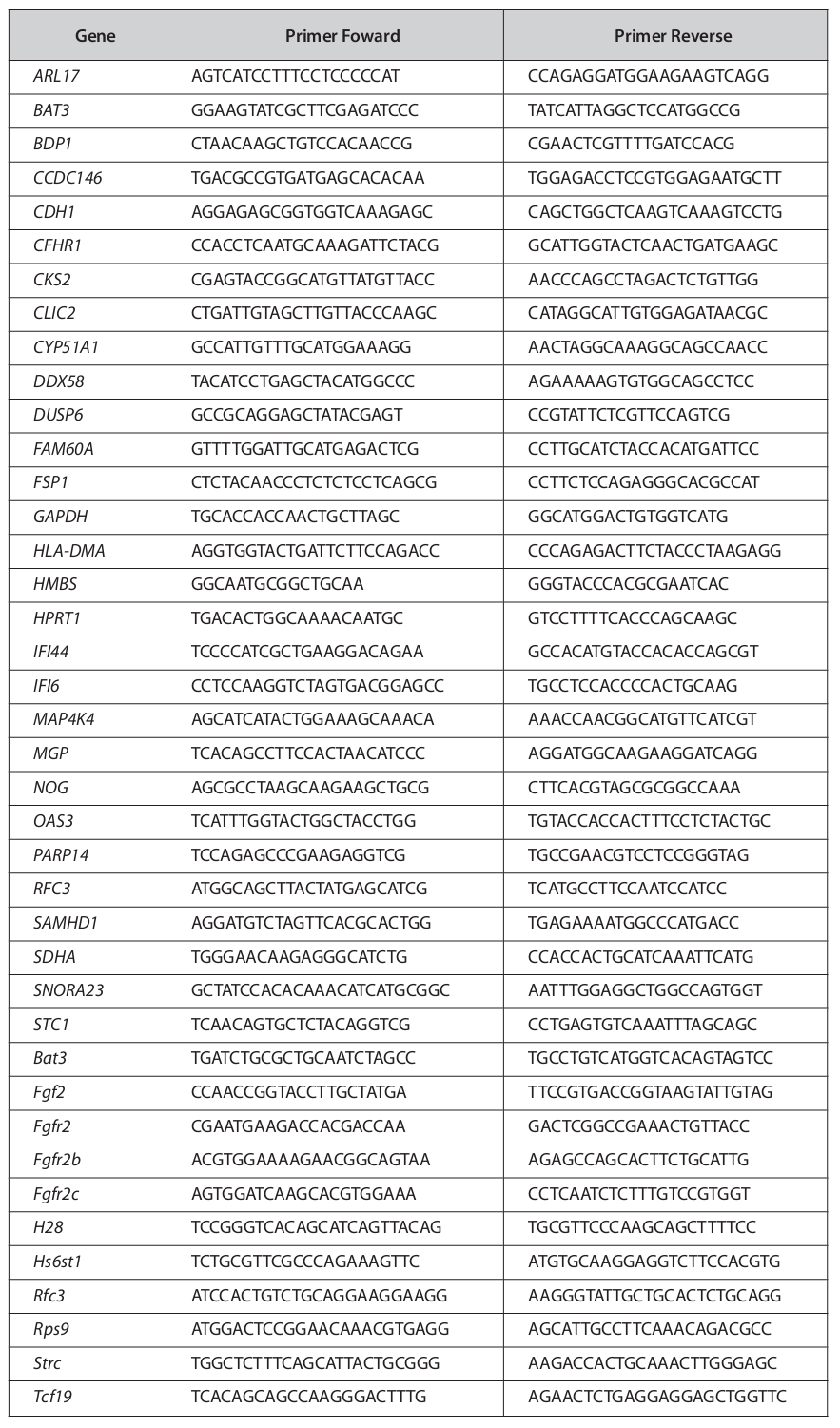

Supplement: Table S1 — Primers used for quantitative real time PCR. (DOCX) [file pone.0060439.s003.docx]
