# Supplementary material for: Novel Molecular Pathways Elicited by Mutant FGFR2 May Account for Brain Abnormalities in Apert Syndrome
Source: PLoS One. 2013 Apr 4;8(4):e60439. doi: 10.1371/journal.pone.0060439 (PMC3617104; doi:10.1371/journal.pone.0060439)
Supplement: Table S2 — Differentially expressed transcripts in FGF2 - treated WT fibroblasts compared to the same samples without treatment. In each treatment, transcripts are ordered by average Fold change ratio (treated vs non-treated) of the replicates. (DOCX) [file pone.0060439.s004.docx]

Table S2

| **Gene Symbol** | **Gene Description** | **Gene Accession** | **Average Fold-Change** |
| --- | --- | --- | --- |
| *C6orf15* | chromosome 6 open reading frame 15 | NM_014070 | **-6.966024851** |
| *LOC100132785* | similar to C21orf99 protein | XM_001720214 | **-3.797081687** |
| *PRY* | PTPN13-like, Y-linked | NM_004676 | **-3.288728118** |
| *IGHV* | immunoglobulin heavy variable group | AF035788 /// AF035797 /// M57945 /// X81730 /// ENST00000390601 /// ENST00000390609 /// ENST00000390624 /// ENST00000390627 /// ENST00000390631 /// ENST00000390632 /// ENST00000390637 | **-2.715496607** |
| *HLA-DMA* | major histocompatibility complex, class II, DM alpha | NM_006120 | **-2.500351849** |
| *RDH10* | retinol dehydrogenase 10 (all-trans) | NM_172037 | **-2.385539686** |
| *SLC39A8* | solute carrier family 39 (zinc transporter), member 8 | NM_022154 | **-2.36555047** |
| *HSD17B6* | hydroxysteroid (17-beta) dehydrogenase 6 homolog (mouse) | NM_003725 | **-2.361945415** |
| *DEPDC6* | DEP domain containing 6 | NM_022783 | **-2.102228375** |
| *ADAMTS5* | ADAM metallopeptidase with thrombospondin type 1 motif, 5 (aggrecanase-2) | NM_007038 | **-2.098701516** |
| *VGLL3* | vestigial like 3 (Drosophila) | NM_016206 | **-2.050439959** |
| *KCNE4* | potassium voltage-gated channel, Isk-related family, member 4 | NM_080671 | **-2.021485471** |
| *ARHGAP28* | Rho GTPase activating protein 28 | NM_001010000 | **-1.972668721** |
| *MSC* | musculin (activated B-cell factor-1) | NM_005098 | **-1.942140455** |
| *DCLK1* | doublecortin-like kinase 1 | NM_004734 | **-1.939175366** |
| *NNMT* | nicotinamide N-methyltransferase | NM_006169 | **-1.93755211** |
| *---* | ncrna:misc_RNA | ENST00000362371 | **-1.904440977** |
| *---* | ncrna:snRNA | ENST00000384227 | **-1.875226794** |
| *PHC1* | polyhomeotic homolog 1 (Drosophila) | NM_004426 | **-1.864107825** |
| *---* | ncrna:rRNA | ENST00000390940 | **-1.861385973** |
| *---* | ncrna:Mt_tRNA_pseudogene | ENST00000385854 | **-1.857462157** |
| *LOC23117* | KIAA0220-like protein | BC094882 | **-1.228017681** |
| *---* | ncrna:misc_RNA | ENST00000364618 | **-1.121662727** |
| *---* | ncrna:scRNA_pseudogene | ENST00000386961 /// ENST00000364899 | **-0.968231174** |
| *---* | ncrna:snRNA_pseudogene | ENST00000388070 | **-0.875481588** |
| *ZNF479* | zinc finger protein 479 | NM_033273 | **-0.873204329** |
| *LOC100130904* | similar to CD177 molecule | XR_037541 /// GENSCAN00000039092 | **-0.749531072** |
| *OCR1* | ovarian cancer-related protein 1 | AF314543 | **-0.683332684** |
| *LOC441728* | similar to Golgin subfamily A member 6 (Golgin linked to PML) (Golgin-like protein) | XR_017329 | **-0.674999649** |
| *---* | ncrna:Mt_tRNA_pseudogene | ENST00000387929 /// ENST00000385593 | **-0.564705734** |
| *TPTE2* | transmembrane phosphoinositide 3-phosphatase and tensin homolog 2 | NM_130785 | **-0.412233497** |
| *---* | --- | ENST00000390891 | **0.007631216** |
| *---* | cdna:pseudogene | ENST00000312946 /// GENSCAN00000054526 | **0.084222544** |
| *---* | ncrna:misc_RNA | ENST00000364153 | **0.087536249** |
| *---* | ncrna:misc_RNA | ENST00000384562 | **0.358227911** |
| *SEPT9* | septin 9 | AK093770 /// AK097965 | **0.436534369** |
| *CYP51A1* | cytochrome P450, family 51, subfamily A, polypeptide 1 | NM_000786 | **0.52657167** |
| *ARP11* | actin-related Arp11 | AB039791 /// ENST00000252071 | **0.584411256** |
| *---* | ncrna:misc_RNA | ENST00000362591 | **0.887119539** |
| *RPL31* | ribosomal protein L31 | NM_001099693 | **1.162937218** |
| *CCDC144C* | coiled-coil domain containing 144C | BC036241 | **1.173107178** |
| *BDP1* | B double prime 1, subunit of RNA polymerase III transcription initiation factor IIIB | NM_018429 | **1.490297658** |
| *DCBLD2* | discoidin, CUB and LCCL domain containing 2 | NM_080927 | **1.788820938** |
| *LOC554202* | hypothetical LOC554202 | AK124391 | **1.789349137** |
| *MAP4K4* | mitogen-activated protein kinase kinase kinase kinase 4 | NM_145686 | **1.805088567** |
| *HIST1H2AB* | histone cluster 1, H2ab | NM_003513 | **1.811556105** |
| *FJX1* | four jointed box 1 (Drosophila) | NM_014344 | **1.900297877** |
| *C11orf41* | chromosome 11 open reading frame 41 | NM_012194 | **1.908718134** |
| *FAM180A* | Family With Sequence Similarity 180, Member A | AK290250 | **1.960976026** |
| *KIAA1462* | KIAA1462 | NM_020848 | **1.971162309** |
| *DTL* | denticleless homolog (Drosophila) | NM_016448 | **1.987433927** |
| *HELLS* | helicase, lymphoid-specific | NM_018063 | **2.012877864** |
| *HHIP* | hedgehog interacting protein | NM_022475 | **2.017011746** |
| *RASA2* | RAS p21 protein activator 2 | NM_006506 | **2.079536598** |
| *CNIH3* | cornichon homolog 3 (Drosophila) | NM_152495 | **2.091466448** |
| *NP* | nucleoside phosphorylase | NM_000270 | **2.14762393** |
| *PTGER2* | prostaglandin E receptor 2 (subtype EP2), 53kDa | NM_000956 | **2.288538348** |
| *CCNE2* | cyclin E2 | NM_057749 | **2.323552122** |
| *SEMA3A* | sema domain, immunoglobulin domain (Ig), short basic domain, secreted, (semaphorin) 3A | NM_006080 | **2.327292666** |
| *ARHGAP22* | Rho GTPase activating protein 22 | NM_021226 | **2.344520903** |
| *CEP170* | Homo sapiens centrosomal protein 170kDa | NM_001042405.1 | **2.409390849** |
| *CDCP1* | CUB domain containing protein 1 | NM_022842 | **2.43881961** |
| *DUSP6* | dual specificity phosphatase 6 | NM_001946 | **2.471975851** |
| *PLAUR* | plasminogen activator, urokinase receptor | NM_002659 | **2.498734816** |
| *SLC20A1* | solute carrier family 20 (phosphate transporter), member 1 | NM_005415 | **2.522113757** |
| *IL13RA2* | interleukin 13 receptor, alpha 2 | NM_000640 | **2.530435058** |
| *LOC204010* | similar to 40S ribosomal protein SA (P40) (34/67 kDa laminin receptor) (Colon carcinoma laminin-binding protein) (NEM/1CHD4) (Multidrug resistance-associated protein MGr1-Ag) | BC107865 | **2.605198537** |
| *FLJ42986* | FLJ42986 protein | AK124976 | **2.632610596** |
| *TFPI2* | tissue factor pathway inhibitor 2 | NM_006528 | **2.753539501** |
| *SHISA3* | shisa homolog 3 (Xenopus laevis) | BC127690 /// BC127691 /// NM_001080505 /// ENST00000319234 | **2.821991676** |
| *CEP170* | centrosomal protein 170kDa | NM_014812 | **2.87472888** |
| *TM4SF1* | transmembrane 4 L six family member 1 | NM_014220 | **2.874756767** |
| *HAS2* | hyaluronan synthase 2 | NM_005328 | **2.908644279** |
| *STC1* | stanniocalcin 1 | NM_003155 | **3.113104009** |
| *---* | ncrna:Mt_tRNA_pseudogene | ENST00000386778 | **3.116975156** |
| *ITGA2* | integrin, alpha 2 (CD49B, alpha 2 subunit of VLA-2 receptor) | NM_002203 | **3.120321882** |
| *LRRC37A2* | leucine rich repeat containing 37, member A2 | NM_001006607 | **4.305719036** |
| *ESM1* | endothelial cell-specific molecule 1 | NM_007036 | **4.348927612** |
| *ZNF714* | zinc finger protein 714 | uc002npn.1 | **3.528910564** |
