# Supplementary material for: Novel Molecular Pathways Elicited by Mutant FGFR2 May Account for Brain Abnormalities in Apert Syndrome
Source: PLoS One. 2013 Apr 4;8(4):e60439. doi: 10.1371/journal.pone.0060439 (PMC3617104; doi:10.1371/journal.pone.0060439)
Supplement: Table S3 — Differentially expressed transcripts in FGF2 - treated S252W fibroblasts compared to the same samples without treatment. In each treatment, transcripts are ordered by average Fold change ratio (treated vs non-treated) of the replicates. (DOCX) [file pone.0060439.s005.docx]

Table S3

| **Gene Symbol** | **Gene Description** | **Gene Accession** | **Average Fold-Change** |
| --- | --- | --- | --- |
| *CDC2L1* | Homo sapiens cell division cycle 2-like 1 (PITSLRE proteins) (CDC2L1), transcript variant 5, mRNA | NM_033489.1 | **-6.508669209** |
| *ZNF714* | zinc finger protein 714 | uc002npn.1 | **-5.015379301** |
| *---* | ncrna:Mt_tRNA_pseudogene | ENST00000388162 /// ENST00000388267 | **-3.562656133** |
| *LOC100132426 /// LOC339562 /// LOC642838 /// LOC100133034 /// LOC644704* | similar to hCG1742442 /// similar to hCG1742442 /// similar to hCG1742442 /// hypothetical LOC100133034 /// similar to hCG1742442 | XM_001715084 /// XM_001713985 /// XM_001714030 /// XR_036886 /// ENST00000377423 /// ENST00000377421 /// ENST00000377415 /// GENSCAN00000055056 /// GENSCAN00000009486 | **-2.469985149** |
| *BDP1* | B double prime 1, subunit of RNA polymerase III transcription initiation factor IIIB | NM_018429 | **-2.246761814** |
| *LOC402182* | hypothetical LOC402182 | XR_016669 | **-1.881510423** |
| *---* | ncrna:snoRNA_pseudogene | ENST00000387663 /// ENST00000386454 | **-1.827255627** |
| *C2* | complement component 2 | NM_000063 | **-1.6494143** |
| *LOC645231* | similar to 60S ribosomal protein L22 (Heparin-binding protein HBp15) | XM_928270 | **-1.100577354** |
| *HS6ST1* | heparan sulfate 6-O-sulfotransferase 1 | NM_004807 | **-1.02876323** |
| *BAT3* | HLA-B associated transcript 3 | NM_004639 | **-0.9104585** |
| *---* | cdna:pseudogene | ENST00000359859 | **-0.876231249** |
| *TBC1D3G* | TBC1 domain family, member 3G | NM_001040282 | **-0.790198544** |
| *SNORD114-2* | small nucleolar RNA, C/D box 114-2 | NR_003194 | **-0.780528593** |
| *---* | Homo sapiens miR-520b stem-loop /// Homo sapiens miR-519a stem-loop | hsa-mir-520b /// hsa-mir-519a-2 | **-0.741233114** |
| *---* | ncrna:misc_RNA | ENST00000391210 | **-0.7211126** |
| *LOC646743* | similar to testicular serine protease 2 | AY726562 | **-0.587339777** |
| *---* | ncrna:scRNA_pseudogene | ENST00000385402 | **-0.584686583** |
| *CYP51A1* | cytochrome P450, family 51, subfamily A, polypeptide 1 | NM_000786 | **-0.336841965** |
| *RSAD2* | radical S-adenosyl methionine domain containing 2 | NM_080657 | **-0.195670965** |
| *IFI44L* | interferon-induced protein 44-like | AL832618 | **0.190567526** |
| *---* | Homo sapiens similar to bA476I15.3 (novel protein similar to septin), mRNA | BC131690 | **0.302541362** |
| *RFC3* | replication factor C (activator 1) 3, 38kDa | NM_002915 | **0.347720151** |
| *PP10897* | Homo sapiens PP10897 | AF370400 | **0.352856713** |
| *NAG13* | Homo sapiens NAG13 mRNA | AF194537 | **0.356798376** |
| *LRRC37A* | leucine rich repeat containing 37A | NM_014834 | **0.402935464** |
| *STRC* | stereocilin | NM_153700 | **0.478896023** |
| *CLDN6* | claudin 6 | NM_021195 | **0.658559518** |
| *PGA3* | pepsinogen 3, group I (pepsinogen A) | NM_001079807 | **0.796611273** |
| *---* | ncrna:Mt_tRNA_pseudogene | ENST00000387490 /// ENST00000387861 /// ENST00000385767 /// ENST00000385604 /// ENST00000385831 | **0.801467771** |
| *---* | ncrna:scRNA_pseudogene | ENST00000386460 | **0.805808129** |
| *C21orf70* | chromosome 21 open reading frame 70 | AF391113 /// AF391114 /// BC009341 /// ENST00000397826 /// ENST00000291634 | **0.958125325** |
| *TCF19* | transcription factor 19 (SC1) | NM_007109 | **1.07050289** |
| *TRNY* | tRNA-Tyr | NC_001807 | **1.079919339** |
| *PRY* | PTPN13-like, Y-linked | NM_004676 | **1.106123871** |
| *---* | ncrna:misc_RNA | ENST00000363257 | **1.198837668** |
| *LY6G6C* | lymphocyte antigen 6 complex, locus G6C | AY359119 | **1.26995483** |
| *---* | ncrna:misc_RNA | ENST00000363265 | **1.309116099** |
| *---* | ncrna:rRNA | ENST00000362694 | **1.337319386** |
| *WDR74* | WD repeat domain 74 | AK292330 | **1.39600724** |
| *---* | ncrna:misc_RNA | ENST00000362996 | **1.586570394** |
| *---* | ncrna:misc_RNA | ENST00000390895 | **1.682492818** |
| *ARHGAP22* | Rho GTPase activating protein 22 | NM_021226 | **1.82344581** |
| *---* | ncrna:misc_RNA | ENST00000390916 | **1.87056716** |
| *---* | ncrna:snRNA | ENST00000383975 | **1.952664313** |
| *---* | ncrna:Mt_tRNA_pseudogene | ENST00000385525 | **1.976838193** |
| *---* | ncrna:misc_RNA | ENST00000365487 | **2.159727483** |
| *---* | cdna:Genscan | GENSCAN00000006634 | **2.164741499** |
| *---* | ncrna:snRNA | ENST00000384592 | **2.407117576** |
| *---* | ncrna:misc_RNA | ENST00000384284 | **2.724788513** |
| *RPS9* | ribosomal protein S9 | NM_001013 | **2.847641991** |
| *---* | ncrna:tRNA_pseudogene | ENST00000386848 | **2.894987738** |
| *---* | --- | --- | **3.607954302** |
| *---* | ncrna:rRNA | ENST00000364003 | **4.356407486** |
| *WDR74 /// RNU2 /// RNU2B* | WD repeat domain 74 /// RNA, U2 small nuclear /// RNA, U2B small nuclear | AK292330 /// NR_002716 /// NR_002761 | **4.37402961** |
